# A cross-sectional study of clinical, dermoscopic, histopathological, and molecular patterns of scalp melanoma in patients with or without androgenetic alopecia

**DOI:** 10.1038/s41598-022-17108-z

**Published:** 2022-09-05

**Authors:** Ana Carolina Porto, Tatiana Pinto Blumetti, Vinícius Fernando Calsavara, Giovana Tardin Torrezan, Cláudia Alessandra Andrade de Paula, Rute Lellis, João Pedreira Duprat Neto, Dirce Maria Carraro, J. Casagrande Tavoloni Braga

**Affiliations:** 1 Cutaneous Oncology Department, A. C. Camargo Cancer Center, Rua Pires da Mota, 1167, São Paulo, SP 01529-001 Brazil; 2 Department of Epidemiology and Statistics, A. C. Camargo Cancer Center, São Paulo, Brazil; 3 Genomics and Molecular Biology Group, A. C. Camargo Cancer Center, Rua Taguá, 440, São Paulo, SP 0508-010 Brazil; 4 National Institute of Science and Technology in Oncogenomics and Therapeutic Innovation, A. C. Camargo Cancer Center, Rua Professor Antonio Prudente, 211, São Paulo, Brazil; 5 Department of Pathology, A. C. Camargo Cancer Center, Rua Professor Antonio Prudente, 211, São Paulo, Brazil

**Keywords:** Melanoma, Melanoma

## Abstract

Scalp melanoma (SM) has a worse prognosis than melanoma in other locations likely because of late diagnosis due to hair coverage, difficulties in interpreting dermoscopy findings, and its unique molecular profile. We aimed to describe the clinical, histopathological, molecular, and dermoscopic patterns of SM and its relation to androgenetic alopecia/elastosis at the tumor site. Through a retrospective cross-sectional study, we identified all SM diagnosed at the A.C.Camargo Cancer Center between 2008 and 2018. In all, 48 SM were analyzed: 45.8% of which exhibited moderate/severe androgenetic alopecia and 54.1% exhibited elastosis. Androgenetic alopecia/elastosis at the site of the SM was associated with older age (*p* < 0.001), chronic sun damage (*p* < 0.001), lentigo maligna subtype (*p* = 0.029), and photodamaged dermoscopic pattern (*p* < 0.001). Additionally, 41 cases were evaluated with a 14-gene panel: 53.7% displayed mutations and 46.3% were wild-type. *BRAF* mutations were most common (77%), with *BRAF* V600K being more frequent (50%) than *BRAF* V600E (31.2%). The *NF1* gene was evaluated in 40 samples, of which 20% exhibited mutations. SM presents differently in areas covered by hair compared to in areas with androgenetic alopecia. Patients without alopecia may have higher Breslow thickness due to late diagnosis because of hair concealment. The high frequency of detrimental mutations can also explain the poor prognosis of SM.

## Introduction

**Figure 1:**
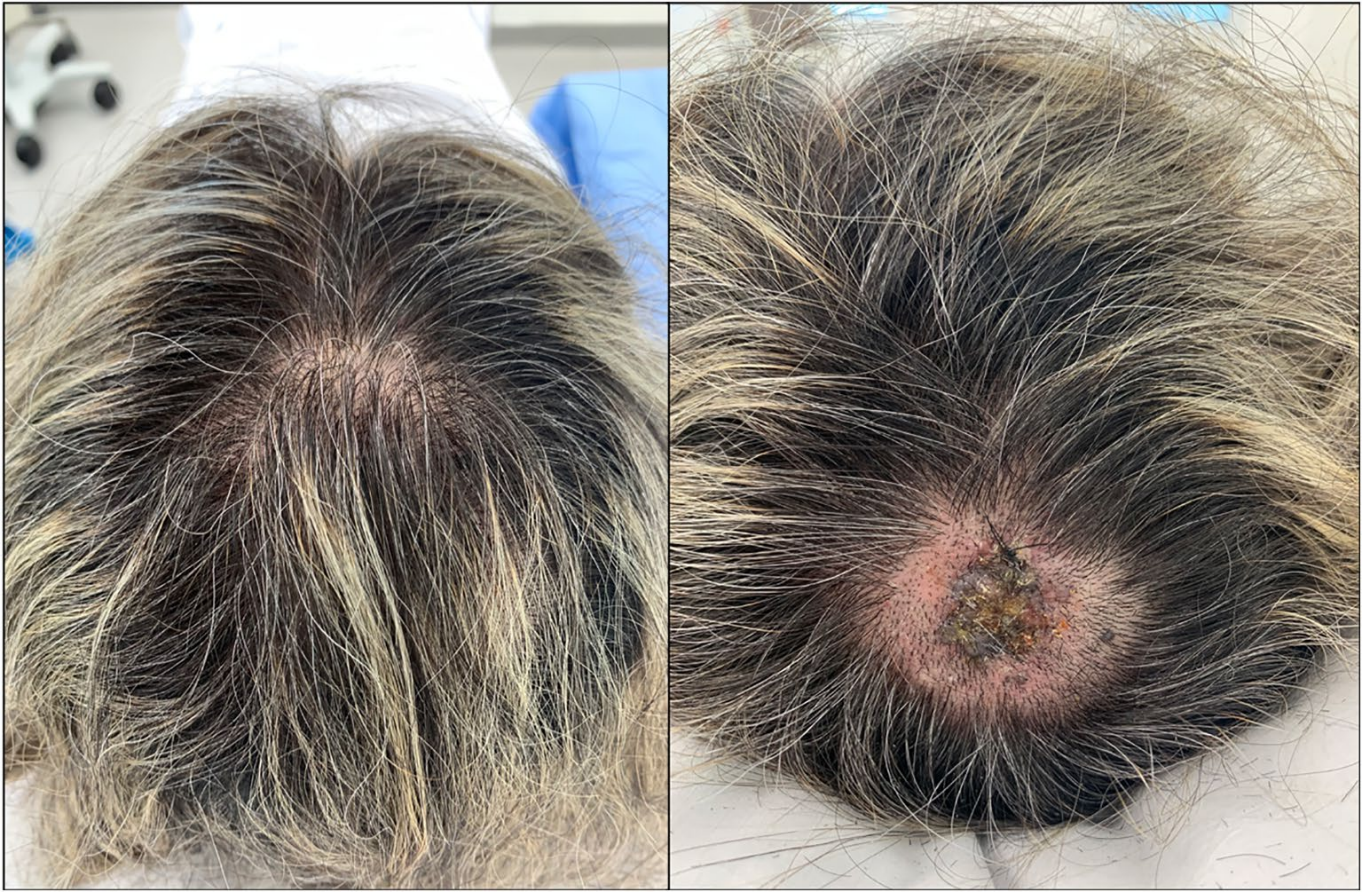
Clinical image of a 57-years-old woman with a melanoma hidden by hair, without androgenetic alopecia or chronic sun damage. Histopathological examination showed a superficial spreading melanoma, with a Breslow thickness of 7.1 mm, without elastosis or signs of androgenetic alopecia.

**Figure 2:**
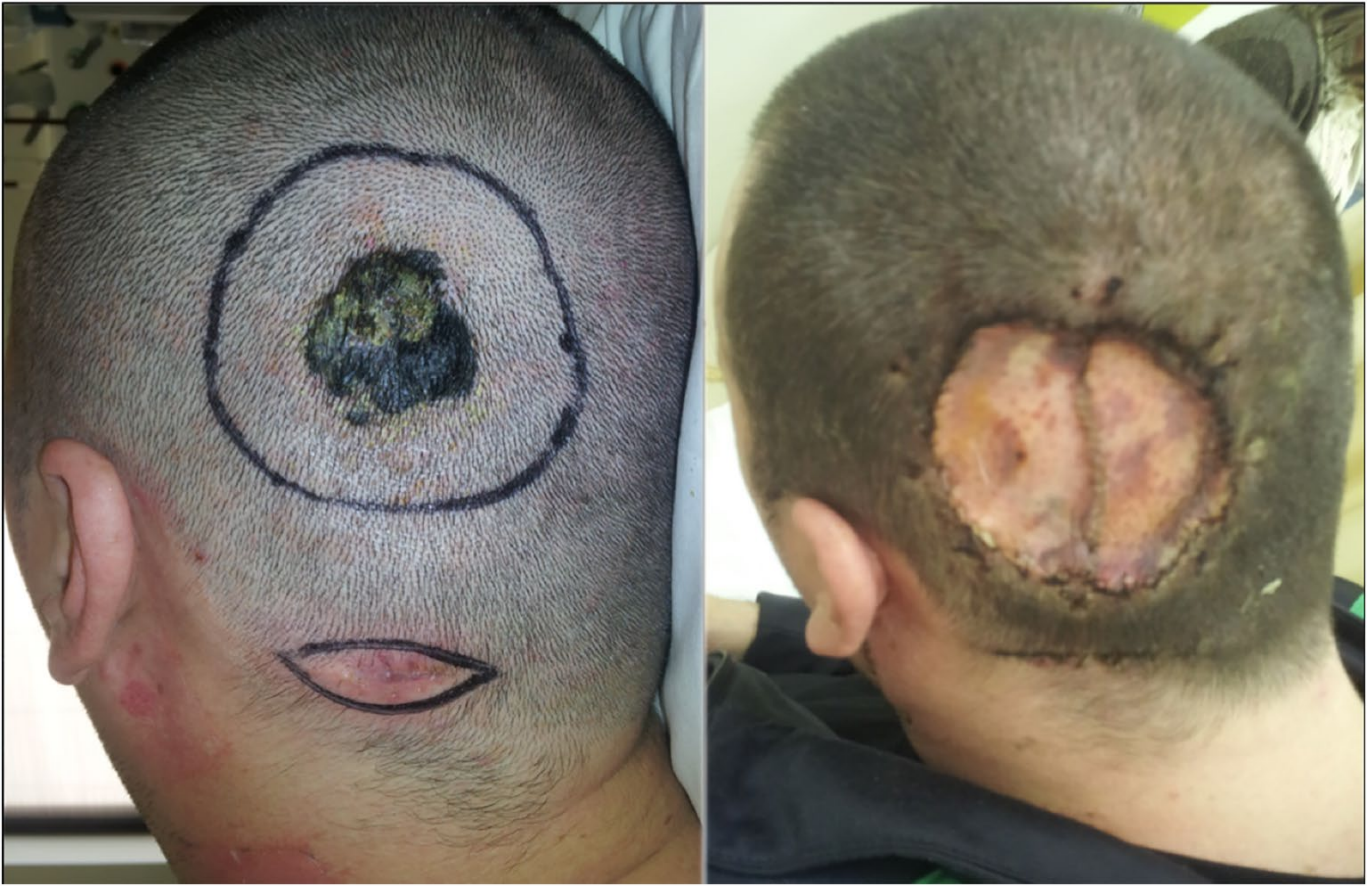
Clinical image of a 28-years-old man with a superficial spreading melanoma, with a Breslow thickness of 3.3 mm. This thick melanoma required complex surgery.

Scalp melanoma (SM) accounts for 5% of all melanomas and tends to occur in older patients with a history of chronic sun damage^1–4^. SM is associated with a greater risk of disease progression and death than melanoma in other sites^5–10^, likely due to hair coverage resulting in later diagnosis (Fig. 1)^11–13^, the high proportion of melanomas with rapid vertical growth^4,9^, the presence of melanomas in an area of higher blood and lymph supply^14,15^, and inadequate surgical margins (Fig. 2)^16^. Although the worse prognosis associated with SM can be attributed to the often high Breslow thickness upon diagnosis, Claeson et al.^10^ demonstrated that the tumor site of the scalp is a strong predictive factor of death, even in patients with thin melanomas.

Melanoma is one of the most frequently mutated cancers. The genetic profile of tumors can determine the risk of recurrence in patients with melanomas^17,18^. Moreover, *BRAF* V600K and *NF1* mutations are associated with older age, higher degrees of chronic sun damage, and lower disease-free survival^19,20^. Thus, the poor prognosis of SM patients could be owing to the higher prevalence of *BRAF* V600K and *NF1* mutations.

Regardless of the tumor site, early diagnosis and subsequent surgical excision are key factors for the favorable prognosis of patients with melanoma. Dermoscopy increases the diagnostic accuracy of melanomas by up to 30%^21^, and although dermoscopic patterns are affected by anatomical location, few studies have described the dermoscopic pattern of SM^22–24^. Understanding the usual presentation of SM can help elucidate the reasons for its poor prognosis. Thus, the aim of this study was to describe the clinical, dermoscopic, histopathological, and molecular profiles of patients with SM.

## Methods

We performed a cross-sectional, retrospective study at the A. C. Camargo Cancer Center, São Paulo, Brazil. The project was approved by the A.C.Camargo Cancer Center Institucional Ethics Committee (2379/17). All included patients provided written informed consent for the publication of their medical details.

We retrieved the electronic medical records of patients diagnosed with SM at the A. C. Camargo Cancer Center, São Paulo, Brazil, from January 2008 to December 2018, per the Declaration of Helsinki. Patients whose dermoscopic images and histopathological data were available in the Skin Center database of our institution were included.

### Clinical data

We collected the following data: sex, age at diagnosis, skin phototype, chronic sun damage to skin defined by a personal history of non-melanoma skin cancer or actinic keratoses, personal and family history of melanoma, and exact anatomical site of occurrence.

### Dermoscopy

Images of the melanomas were obtained using a dermatoscope with polarized light (Dermlitell ProHR) attached to a digital camera. Dermoscopic criteria were selected for assessment based on existing literature. We evaluated 10 melanoma-specific structures (classic dermoscopic pattern) in the lesions as well as 10 melanoma-specific structures found in facial and nonfacial sun-damaged skin (photodamaged dermoscopic pattern)^25–27^.

The presence of androgenetic alopecia in the skin affected by melanoma was evaluated using the following dermoscopic characteristics: hair shaft thickness heterogeneity, brown peripilar sign, white peripilar sign, yellow dots, pinpoint white dots, focal atrichia, and scalp pigmentation^28^. Dermoscopic images were evaluated by three independent investigators (JCT, TPB, and ACP), blinded to the histopathological diagnosis. For statistical analysis, we classified patients into two groups: (1) without androgenetic alopecia, defined as those with mild or no androgenetic alopecia and (2) with androgenetic alopecia, defined as those with moderate to severe androgenetic alopecia.

### Histopathology

An experienced dermatopathologist subtyped the histological melanoma specimens based on Breslow thickness, presence of ulceration, mitosis, perineural invasion, and associated nevus.

The presence of androgenetic alopecia in the skin affected by melanoma was determined using the following microscopic characteristics: number of follicles and terminal threads, follicular miniaturization, and fibrous tracts below the miniaturized threads. The histopathological evaluation of androgenetic alopecia was compared with the dermoscopic evaluation of androgenetic alopecia, and the classification was made based on consensus.

Chronic solar damage was measured according to the degree of solar elastosis of the skin affected by the melanoma using the 11 criteria previously published by Landi et al.^29^. We considered a chronic solar damage level of 0 to − 2 as the absence of elastosis and a level of ≥ 2 as the presence of elastosis.

### Somatic mutation analysis

Formalin-fixed paraffin-embedded tissues containing at least 30% of tumor cells were selected, and genomic DNA was extracted using a QIAamp DNA FFPE Tissue Mini Kit (Qiagen, Hilden, Germany) following the standard protocol prescribed in the A. C. Camargo Cancer Center Biobank. Targeted next-generation sequencing was performed using two custom Ion AmpliSeq Panels (Thermo Fisher Scientific, Waltham, MA, USA). The first panel covered hotspot regions of 14 genes frequently mutated in melanomas (*BRAF*, *EGFR*, *ERBB2*, *HRAS*, *IDH1*, *IDH2*, *JAK2*, *KIT*, *KRAS*, *MET*, *NRAS*, *PDGFRA*, *PIK3CA*, and *ROS1*). The second customized panel covered 97.4% of the coding region of *NF1*. For each panel, 20 ng of DNA was treated with UDG enzyme (Thermo Fisher Scientific)^30^ and submitted for multiplex amplification using the Ion AmpliSeq Library Kit 2.0 (Thermo Fisher Scientific). The DNA was then sequenced on the Ion Proton platform (Thermo Fisher Scientific) according to the manufacturer’s instructions. The resulting reads were mapped, and variant calling was performed using the Torrent Suite Browser and Torrent Variant Caller software (Thermo Fisher Scientific). Somatic mutations were defined as variant alleles present in more than 2% of the reads with a minimum coverage of 100×.

### Statistical analysis

To evaluate the association between two qualitative variables, Fisher’s exact test or the chi-square test was applied. Comparison between two means (independent groups) was performed using Student’s *t*-test when the assumptions of normality were satisfied; otherwise, the nonparametric Mann–Whitney U test was applied. The Shapiro–Wilk test was used to evaluate the sample distribution. In addition, we fitted a generalized linear model with Poisson and negative binomial distributions and a log link function to the dataset to evaluate the effect of independent variables on the outcomes of classic dermoscopic patterns and photodamaged dermoscopic patterns. Statistical analyses were performed using R software version 3.5 (R Foundation for Statistical Computing, Vienna, Austria). Statistical significance was set at *p* < 0.05 for all tests.

## Results

From January 2008 to December 2018, 154 patients were diagnosed with SM at our institution. Of them, 48 had dermoscopic images and histopathological data available at the Skin Center. Patient characteristics according to the presence of androgenetic alopecia and elastosis are summarized in Table 1.

**Table 1:** Clinical and histopathological characteristics of scalp melanoma patients according to the presence of androgenetic alopecia.

Variable	Alopecia (no)	Alopecia (yes)	*p*	Elastosis (no)	Elastosis (yes)	*p*
**Sex, n (%)**						
Male 0	18 (69.2)	20 (90.9)	0.084*	13 (59.1)	25 (96.2)	0.003*
Female 1	8 (30.8)	2 (9.1)		9 (40.9)	1 (3.8)	
**Age (years)**						
Mean ± SD	52.27 ± 17.20	73.18 ± 9.03	< 0.001**	50.81 ± 18.16	71.19 ± 9.89	< 0.001**
Median (min–max)	56.50 (20.0–78.0)	74.50 (57.0–86.0)		55 (20.0–86.0)	73 (45–86)	
**Chronic sun damage, n (%)**						
No	19 (76)	2 (9.1)	< 0.001****	15 (71.4)	6 (23.1)	0.003****
Yes	6 (24)	20 (90.9)		6 (28.6)	20 (76.9)	
**History of melanoma, n (%)**						
No	18 (69.2)	17 (77.3)	0.765****	14 (63.6)	21 (80.8)	0.315****
Yes	8 (30.8)	5 (22.7)		8 (36.4)	5 (19.2)	
**Family history of melanoma, n (%)**						
No	21 (80.8)	21 (95.5)	0.199*	17 (77.3)	25 (96.2)	0.081*
Yes	5 (19.2)	1 (4.5)		5 (22.7)	1 (3.8)	
**Skin phototype, n (%)**						
1	0 (0)	1 (4.8)	0.617*	0 (0)	1 (4.2)	0.999*
2	18 (75)	14 (66.7)		15 (71.4)	17 (70.8)	
3	6 (25)	6 (28,6)		6 (28.6)	6 (25)	
**Localization, n (%)**						
Parietal	12 (48)	13 (65)	0.045*	10 (47.6)	15 (62.5)	0.200*
Frontal	0 (0)	4 (20)		1 (4.8)	3 (12.5)	
Vertex	6 (24)	2 (10)		5 (23.8)	3 (12.5)	
Occipital	3 (12)	1 (5)		1 (4.8)	3 (12.5)	
Nuchal	1 (4)	0 (0)		1 (4.8)	0 (0)	
Temporal	3 (12)	0 (0)		3 (14.3)	0 (0)	
**Tumor subtype, n (%)**						
Superficial spreading	23 (88.5)	11 (52.4)	0.001*	20 (90.9)	14 (56.0)	0.003*
Nodular	1 (3.8)	1 (4.8)		0 (0)	2 (8.0)	
Lentigo maligna	0 (0)	7 (33.3)		0 (0)	7 (28.0)	
Desmoplastic	1 (3.8)	0 (0)		1 (4.5)	0 (0)	
Other	1 (3.8)	2 (9.6)		1 (4.5)	2 (8.0)	
**Breslow thickness (mm)**						
Mean ± SD	1.53 ± 2.09	1.34 ± 2.75	0.186***	1.65 ± 2.45	1.27 ± 2.38	0.190***
Median (min–max)	0.6 (0–7.1)	0 (0–9.6)		0.7 (0–9.6)	0.15 (0–9)	
**Associated nevus, n (%)**						
No	21 (80.8)	17 (77.3)	0.999*	16 (72.7)	22 (84.6)	0.47*
Yes	5 (19.2)	5 (22.7)		6 (27.3)	4 (15.4)	
**Ulceration, n (%)**						
No	23 (88.5)	21 (95.5)	0.614*	20 (90.9)	24 (92.3)	0.999*
Yes	3 (11.5)	1 (4.5)		2 (9.1)	2 (7.7)	
**Mitosis, n (%)**						
No	17 (65.4)	18 (81.8)	0.202****	14 (63.6)	21 (80.8)	0.183****
Yes	9 (34.6)	4 (18.2)		8 (36.4)	5 (19.2)	
**Perineural invasion, n (%)**						
No	26 (100)	20 (90.9)	0.205*	21 (95.5)	25 (96.2)	0.999*
Yes	0 (0)	2 (9.1)		1 (4.5)	1 (3.8)	
**Elastosis, n (%)**						
No	20 (76.9)	2 (9.1)	< 0.001****			
Yes	6 (23.1)	20 (90.1)				
**Classic dermoscopic pattern**						
Mean ± SD	2.54 (1.28)	2.5 (1.70)	0.828***	2.55 (1,27)	2.5 (1.64)	0.81***
Median (min–max)	2 (1–5)	2 (0–6)		2 (1–5)	2 (0–6)	
**Photodamaged dermoscopic pattern**						
Mean ± SD	1.04 ± 1.75	2.95 ± 1.93	< 0.001***	0.55 ± 0.99	3.04 ± 2.03	< 0.001***
Median (min–max)	0 (0–7)	2.5 (0–8)		0 (0–4)	3 (0–8)	
***BRAF* V600K**						
No	15 (75)	15 (83.3)	0.697*	13 (81.2)	17 (77.3)	0.999*
Yes	5 (25)	3 (16.7)		3 (18.8)	5 (22.7)	
***BRAF* V600E**						
No	15 (75)	18 (100)	0.048*	11 (68.8)	22 (100)	0.009*
Yes	5 (25)	0 (0)		5 (31.2)	0 (0)	
***KIT***						
No	19 (95)	17 (94.4)	0.999*	15 (93.8)	21 (95.5)	0.999*
Yes	1 (5)	1 (5.6)		1 (6.2)	1 (4.5)	
***NF1***						
No	10 (66.7)	19 (90.5)	0.103*	8 (61.5)	21 (91.3)	0.073*
Yes	5 (33.3)	2 (9.5)		5 (38.5)	2 (8.7)	
***NRAS***						
No	20 (100)	16 (88.9)	0.218*	16 (100)	20 (90.9)	0.499*
Yes	0 (0)	2 (11.1)		0 (0)	2 (9.1)	

*Fisher’s exact test; **Student’s t-test; ***Mann–Whitney test; ****Chi-square test.

### Clinical data

The mean age was 61.8 years, and patients with androgenetic alopecia (mean 73.18) or elastosis (mean 71.19) tended to be older than patients without androgenetic alopecia (mean 52.26) or elastosis (mean 50.81). Although most patients were men (79.16%), androgenetic alopecia (*p* = 0.084) occurred at similar frequencies in both sexes, while a higher proportion of men presented with elastosis (*p* = 0.003). Additionally, Fitzpatrick skin II was the most common phototype in this study, for patients with (66.7%) and without androgenetic alopecia (75.0%) as well as those with (71.4%) and without elastosis (71.4%). SMs were predominantly located in the parietal area of the scalp (55.6%). There was no difference in the location of elastosis (*p* = 0.200); however, a difference in the location of androgenetic alopecia was noted (*p* = 0.045).

Chronic sun damage to the skin was significantly more common in patients with androgenetic alopecia and elastosis than in patients without androgenetic alopecia and elastosis (*p* < 0.001 and *p* = 0.003, respectively). Personal and family history of melanoma occurred with similar frequency in all patients, irrespective of androgenetic alopecia and elastosis status.

### Histopathology revealed that SM subtypes were associated with hair loss and elastosis

Histopathological evaluation revealed 20 in situ and 28 invasive melanomas. Eight (40%) of the in situ melanomas were classified as the lentigo maligna (LM) subtype. Moreover, Breslow thickness was slightly higher in patients without androgenetic alopecia than in those with androgenetic alopecia (mean ± SD: 1.53 ± 2.09 mm vs. 1.34 ± 2.75 mm, respectively). Similarly, patients without elastosis had higher Breslow thickness than patients with elastosis (mean ± SD: 1.65 ± 2.45 mm vs. 1.27 ± 2.38 mm, respectively), although the difference was not significant (*p* = 0.186 and *p* = 0.190, respectively). Ulceration, mitosis, perineural invasion, and associated nevus occurred at similar frequencies in patients with or without androgenetic alopecia and elastosis. Histopathologically, tumors were classified as superficial spreading (75%), LM (16.6%), nodular (4.3%), desmoplastic (2.0%), and unclassifiable melanoma (2.0%). The frequency of the LM subtype was strongly associated with the presence of androgenetic alopecia and elastosis.

### Somatic mutation analysis revealed that *BRAF* mutations were predominant in SM

Somatic mutations were investigated in 50 melanoma samples from 48 patients; 5 samples were excluded owing to low quality of next-generation sequencing data. We obtained results for the 14-gene and *NF1* complete gene panel testing of 36 samples, while we only obtained results for the 14-gene panel testing of 5 samples and for the *NF1* panel testing of 4 samples. Of the 41 samples evaluated with the 14-gene panel, 22 (53.7%) displayed at least one mutation, while 19 were wild-type (46.3%). Of the mutations, 17 (77%) were in *BRAF*, 2 (9%) in *NRAS*, 2 (9%) in *KIT*, 1 (4.5%) in *MET*, 1 (4.5%) in *IDH1*, and 1 (4.5%) in *PIK3CA*. Most patients harbored mutations in only one gene, while 8 (36.3%) patients presented mutations in more than one gene.

*BRAF* mutations were detected in 16 melanomas, of which 50% were valine (V) to lysine (K) substitutions in codon 600, 31.2% were V to glutamic acid (E) substitutions in codon 600, and 18.8% were other *BRAF* hotspot mutations. *BRAF* V600E mutations occurred only in the absence of androgenetic alopecia (*p* = 0.048) and elastosis (*p* = 0.009). *BRAF* V600K occurred at a similar frequency regardless of the presence of androgenetic alopecia (*p* = 0.697) or elastosis (*p* = 0.999).

*NRAS* mutations occurred in two melanomas, both in the presence of androgenetic alopecia and elastosis. Finally, *NF1* mutations occurred in eight melanomas.

### Dermoscopic results indicated that Breslow thickness of SMs was associated with the presence of hair in the affected region

The dermoscopic characteristics of SMs are summarized in Supplement Table 1. The classic dermoscopic pattern was observed irrespective of androgenetic alopecia (*p* = 0.828) or elastosis (*p* = 0.81) status. Meanwhile, the photodamaged dermoscopic pattern was significantly more frequent in patients with androgenetic alopecia and elastosis (*p* < 0.0001). In patients with androgenetic alopecia, the most common features were regression structures (66.7%), perifollicular granularity (61.1%), and diffuse/multifocal hypopigmentation (61.1%). In patients without androgenetic alopecia, the most common features were regression structures (45.2%), atypical vessels (41.9%), and an atypical network (38.7%). Thin melanomas (Breslow thickness < 1 mm) were more frequently associated with regression structures (58.3%), atypical networks (47.2%), diffuse/multifocal hypopigmentation (44.4%), and perifollicular granularity (44.4%), whereas thick melanomas (Breslow thickness > 1 mm) were more frequently associated with atypical vessels (53.8%), regression structures (38.5%), and bluish white veils (30.8%).

According to the simple generalized linear model estimates (Supplement Table 2), patients with androgenetic alopecia had a mean photodamage pattern score almost three times higher than that of patients without androgenetic alopecia (2.823, 95% CI 1.487–5.357). Furthermore, the mean photodamage pattern score was nearly 4% higher in older patients (1.038, 95% CI 1.016–1.062). For each 0.1 mm increase in the Breslow thickness, there was a nearly 20% reduction in the photodamage pattern score (0.817, 95% CI 0.668–0.999). The presence of mitosis also reduced the mean score by 76% (0.233, 95% CI 0.089–0.612). Finally, the presence of classic dermoscopic criteria did not depend on age, sex, presence of androgenetic alopecia, mitosis, perineural invasion, associated nevus, Breslow thickness, or gene profile.

## Discussion

Several population studies have shown that SM is more frequent in elderly patients with a mean age of 65 years, which is nearly 10 years older than the age of patients with melanoma located in the trunk or limbs^31^. The mean age of patients with SM in the present study was slightly lower than that described in the literature, which is at 61.85 years. These results could be explained by the demographic characteristics of Brazil, which has a population with a lower life expectancy than Europe and North America^32^. Additionally, the mean age was higher of patients with androgenetic alopecia (mean 73.18) than of those without androgenetic alopecia (mean 52.26).

In line with previous studies, SM occurred six times more frequently in men than in women^4,33,34^. Furthermore, SM was associated with chronic sun damage but not with skin phototype or personal and family history of melanoma. Previous studies proposed that the hair shaft provides physical scalp protection against UV radiation^35^ and we observed that androgenetic alopecia was significantly associated with elastosis and a dermoscopic pattern of photodamage. Androgenetic alopecia is typically more frequent in men; however, in the present study, we detected an equal frequency of the disease in SM patients of both sexes, reinforcing its importance as a risk factor for this type of tumor.

**Figure 3:**
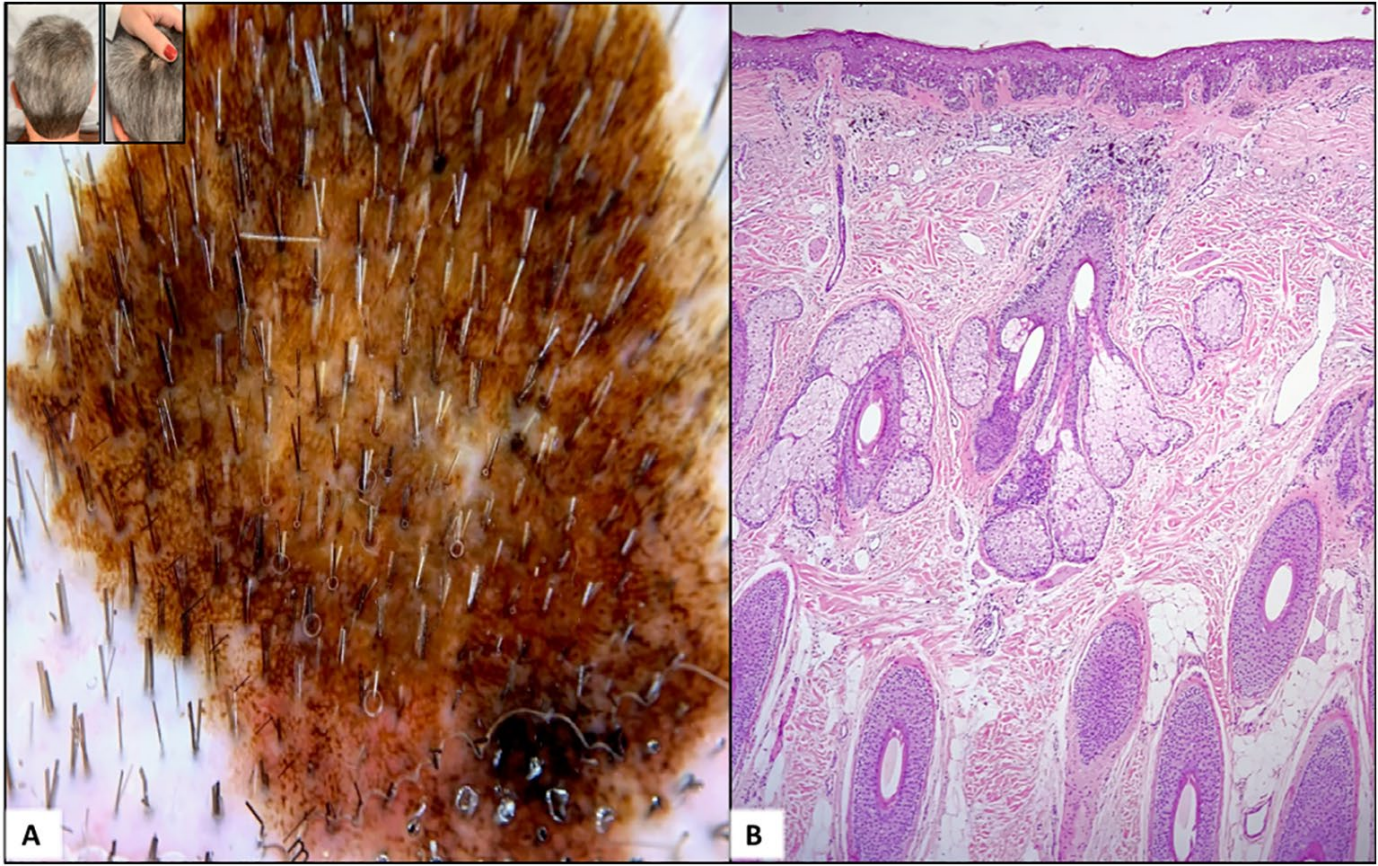
Case of a 54-years-old man with a melanoma hidden by hair, without androgenetic alopecia or chronic sun damage; (**A**) Dermoscopic image of the lesion showing atypical network and globules, off-center blotch, regression structures, and atypical vessels; (**B**) Histopathological examination showing a superficial spreading melanoma, with a Breslow thickness of 0.4 mm, without elastosis or signs of androgenetic alopecia.

**Figure 4:**
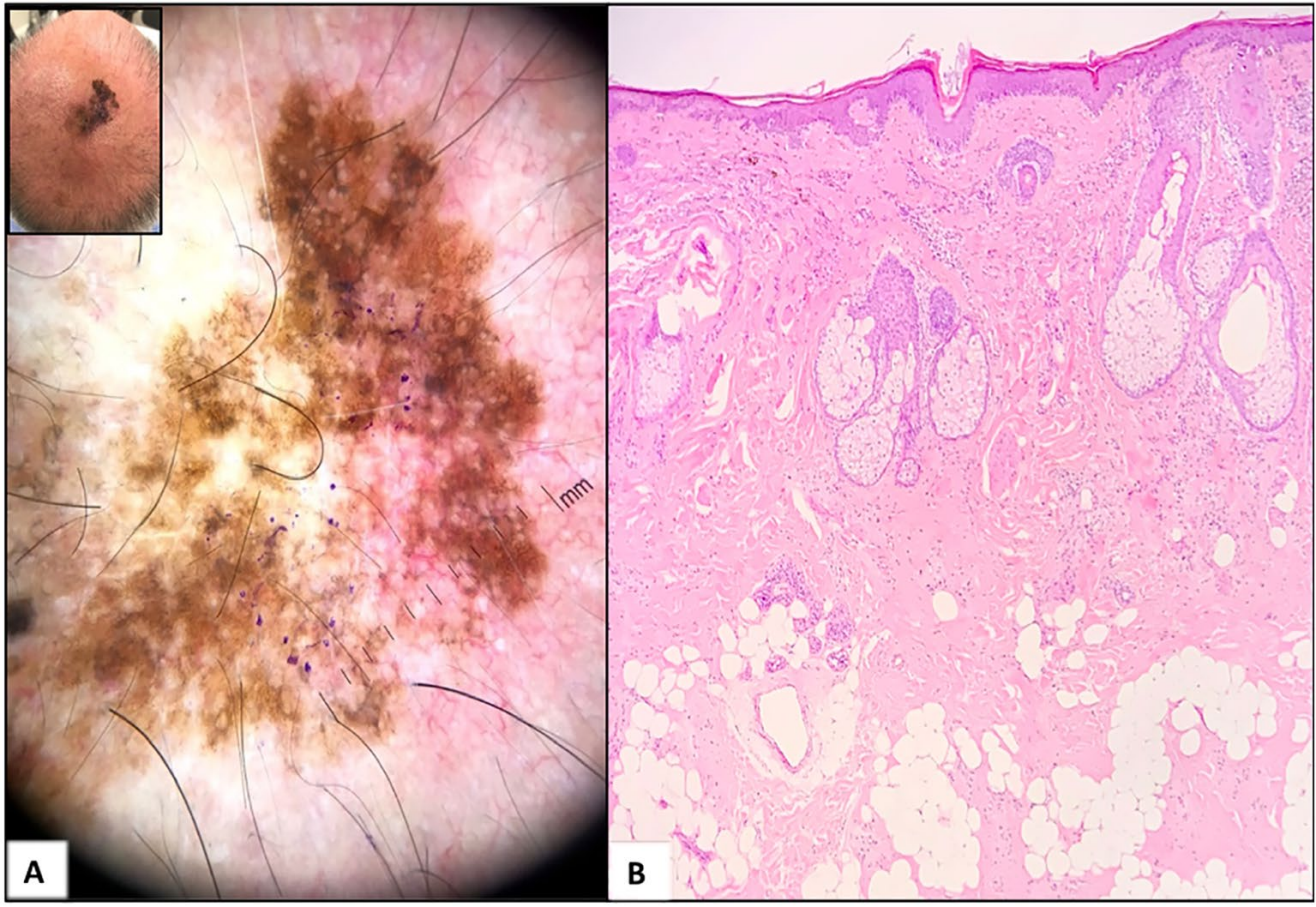
Case of a 58-years-old man with severe androgenetic alopecia and an atypical melanocytic lesion in an area without hair; (**A**) Dermoscopic image showing a classic dermoscopic pattern (regression structures and peripheral tan structureless areas) and a photodamaged dermoscopic pattern (perifollicular granularity; gray, asymmetric perifollicular openings; polygonal and rhomboidal structures; follicle obliteration; concentric circles; and angulated lines); (**B**) Histopathological examination showing a lentigo maligna with severe elastosis and signs of androgenetic alopecia on the tumor surface.

Because most SM studies are retrospective, a precise diagnosis of androgenetic alopecia is not always feasible. Additionally, in some cases, patients may have severe androgenetic alopecia yet develop melanoma in areas covered by hair that are not exposed to chronic sun damage (Fig. 3). Therefore, we evaluated histopathological criteria suggestive of androgenetic alopecia and elastosis at the site of the tumor, aiming to assess whether it is an area of chronic solar damage. According to this evaluation, 45.8% of patients had moderate to severe androgenetic alopecia and 54.1% had moderate to severe elastosis at the tumor site (Fig. 4).

**Figure 5:**
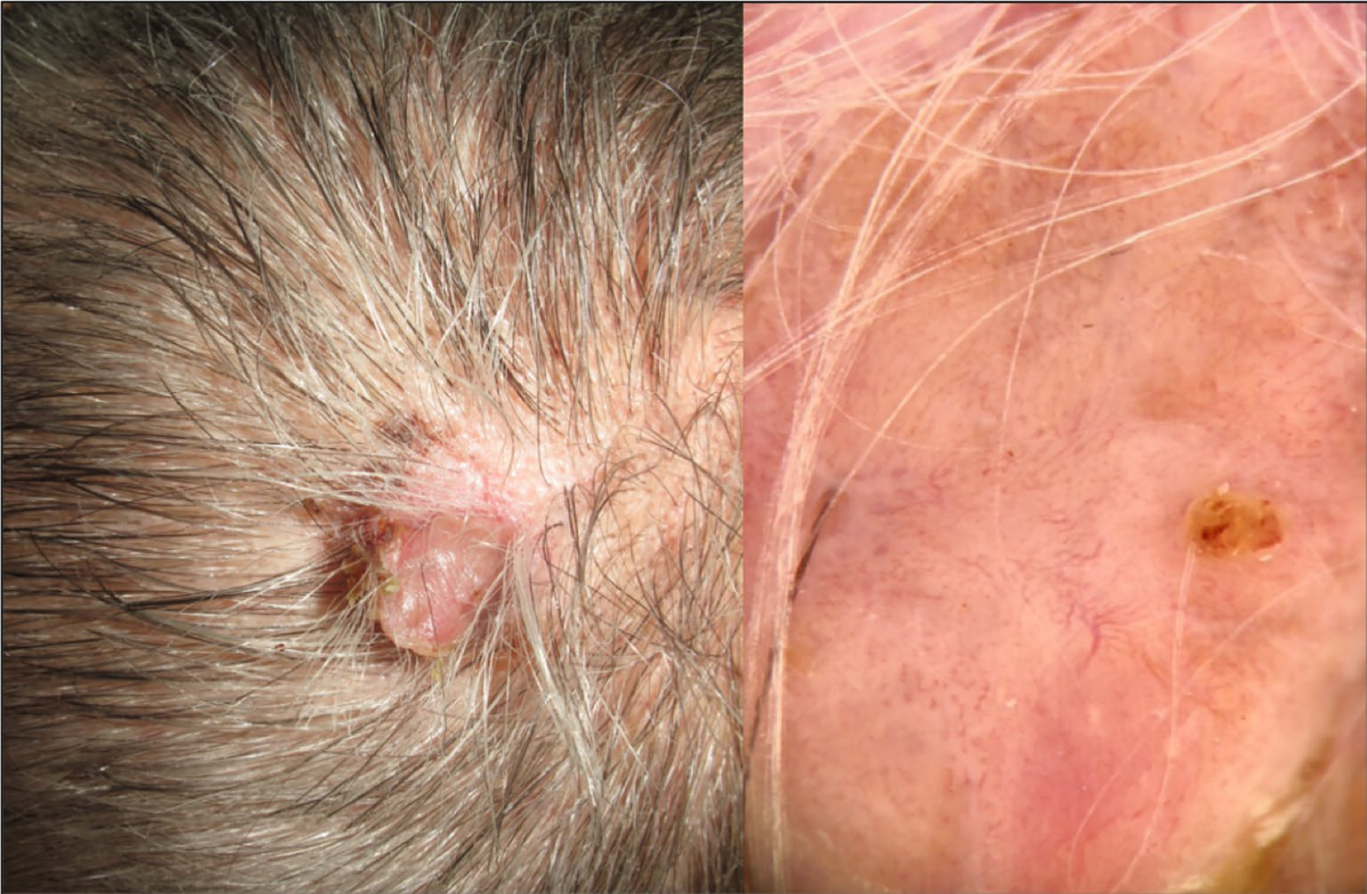
(**A**) Clinical image of a 74-years-old man with an amelanotic papule in the scalp vertex; (**B**) Dermoscopic image of the lesion showing polymorphous vessels and ulceration. Histopathological examination showed a nodular melanoma with a Breslow thickness of 5.5 mm.

**Figure 6:**
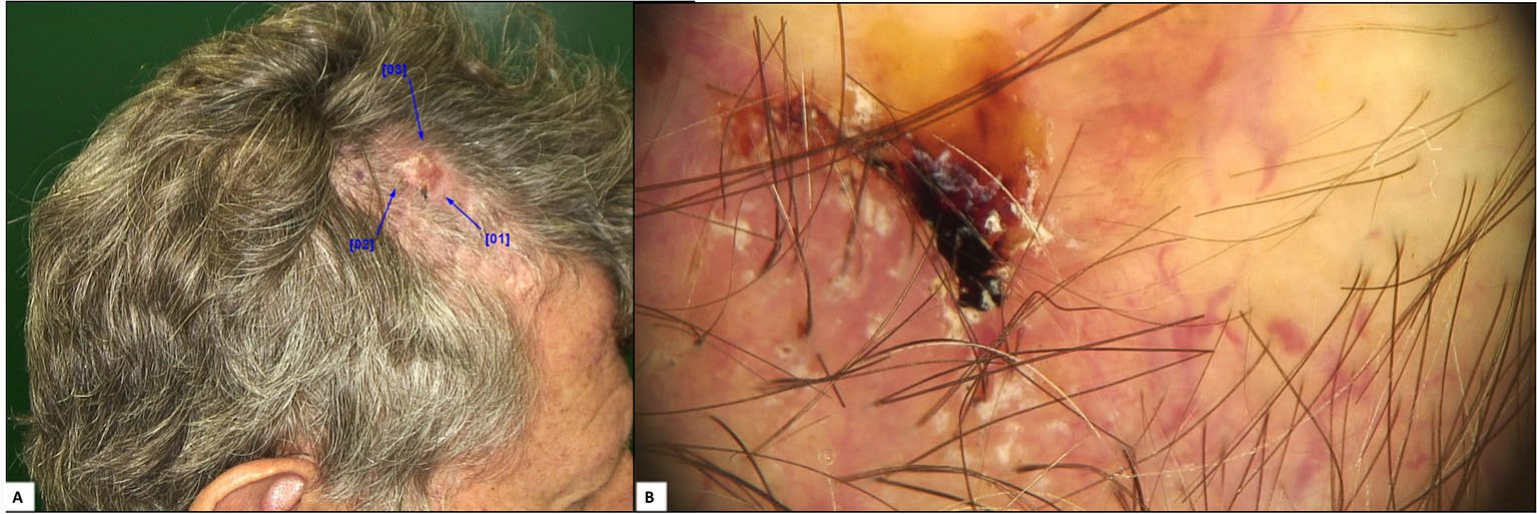
(**A**) Clinical image of a 71-years-old man with a poorly delimited amelanotic plaque; (**B**) Dermoscopic image of the lesion showing polymorphous vessels and ulceration. Histopathological examination showed a desmoplastic melanoma throughout the entire skin thickness and invasion of the skull on diagnosis.

SM is frequently diagnosed with high Breslow thickness^6^, likely owing to late diagnosis because the tumor is hidden under the hair. Nevertheless, androgenetic alopecia is highly prevalent in SM patients^4,35^ with a rate of nearly 50% in the present study. Additionally, we observed slightly thicker SMs in patients without androgenetic alopecia, strengthening the hypothesis that high Breslow thickness is due to the tumor being concealed under the hair. Another possible cause of the higher Breslow thickness in SM is the higher proportion of aggressive subtypes^36,37^. Of the 48 cases we observed, 4.1% were nodular melanoma (Fig. 5) and 2% were desmoplastic melanoma (Fig. 6), which are consistent with the rates of these subtypes of melanoma in other sites^38–41^.

*BRAF*-mutated melanoma represents approximately 50% of all melanomas^42^ and is associated with younger age, lack of elastosis at the tumor site, and truncal location^19,43^. Generally, SM comprises head and neck melanomas and is associated with solar elastosis and less frequently with *BRAF* mutations^44,45^. Despite this tendency, 41.5% of the SMs in our cohort were *BRAF*-mutated. In general, the *BRAF* V600 gain-of-function mutation is most common from V to E (70%), V to K (10–30%), or V to R (5%)^46^. In contrast, the present study showed different frequencies of these mutations: 50% was V–K, 31.2% was V–E, and 18.8% was other *BRAF* mutation locations. Patients with *BRAF* V600K mutations tend to be older and have more chronic sun damage^47^. Some evidence suggests that these patients have a significantly shorter disease-free survival and overall survival^19^. The high frequency of *BRAF* V600K mutations observed in SM can partially explain the unique behavior of this subgroup of melanomas.

*KIT* mutations are reported in 3% of melanomas and are more common in acral and mucosal sites as well as sun-exposed skin melanomas. Moreover, *NRAS* mutations are associated with congenital melanocytic nevi^43^. Both*, KIT* and *NRAS* mutations occur in approximately 4.8% of SM cases.

*NF1* is a tumor suppressor gene, and its loss-of-function has been described in up to 10–15% of melanomas. This mutation is more common in chronically sun-exposed skin of older male patients^48^. *NF1* mutation is associated with poor survival outcomes in patients with melanoma, as well as those with other cancers, such as breast cancer and head and neck squamous cell carcinoma^20^. Although identification of *NF1* mutations is challenging owing to the size and complexity of the gene, we observed these mutations in 20% of SMs, which is a possible explanation for the worse prognosis of patients with SM.

Regardless of location, early diagnosis with dermoscopy increases the chances of a favorable prognosis for patients with melanoma^21^. The scalp is a frequently neglected region during dermatological examination and is difficult for patients to self-examine. Additionally, identifying melanomas hidden under the hair or interspersed with other benign, pigmented lesions in a chronically sun-damaged bald scalp is challenging^26,33^.

Until 2012, only two case reports described dermoscopic patterns of SM. The first report described a pattern similar to that of trunk and limb melanomas^22^, while the second referred to a pattern similar to that of facial melanomas^23^. In 2012, Stanganelli et al*.* described the dermoscopic patterns of 71 SMs. They found that regression and atypical pseudo-networks were more common in thin melanomas, whereas irregular pigmented blotches and unspecific patterns were more common in thick lesions. The authors did not find a correlation between dermoscopic pattern and androgenetic alopecia^24^. In our study, the most frequent dermoscopic finding was regression structures (53%), followed by an atypical network, diffuse or multifocal hypopigmentation, and perifollicular granularity (38.7%, each). However, we observed that androgenetic alopecia was associated only with the photodamaged dermoscopic pattern.

The high dermoscopic variability reported in SM is likely owing to the variety of histopathological subtypes of melanoma in the scalp area: LM especially in patients with alopecia and chronic sun-damaged skin; superficial spreading melanoma in patients with a hairy scalp; and nodular and desmoplastic melanomas. The last two subtypes are rare but most commonly found on the scalp^31^.

In the Stanganelli et al.^24^ publication, the authors did not find a correlation between androgenetic alopecia and Breslow thickness, while Benati et al.^33^ found thicker melanomas in bald patients than in those with hair. Garbarino et al.^49^ analyzed 97 flat SMs and found that superficial spreading melanoma was most often invasive while the LM subtype was most often in situ. The authors hypothesized that LM demonstrates slower growth and easier diagnosis because it is visible to clinical examination^49^. In our study, melanomas in patients with androgenetic alopecia and elastosis were associated with lower Breslow thickness. These findings suggest that a higher Breslow thickness in SM is related to late diagnosis and the tumor’s concealment under the hair.

In conclusion, SM has distinct clinical, dermoscopic, histopathological, and molecular profiles in areas with or without androgenetic alopecia. SM in areas with androgenetic alopecia and elastosis occurs more frequently in elderly patients with chronic sun damage, presenting as the LM subtype, and without *BRAF* V600E mutation than SM without androgenetic alopecia and elastosis. A simple, generalized linear model showed that SM in areas with androgenetic alopecia and elastosis is more likely to present with photodamaged dermoscopic patterns and have lower Breslow thickness and absence of mitosis. The lower Breslow thickness in SM patients with androgenetic alopecia could be owing to easier tumor visualization and earlier diagnosis, in contrast to tumors hidden under the hair. Thus, proactive screening of scalp areas should be encouraged.

Finally, SM has a distinct molecular profile with a high frequency of *BRAF* V600K and *NF1* mutations. These adverse prognostic markers can explain the unique behavior of this subgroup of melanomas. Further studies are necessary to validate these findings.

## Supplementary Information

Supplementary Table 1.

Supplementary Table 2.

## Data Availability

Sequencing data was submitted to the European Variation Archive (Project: PRJEB53894; Analyses: ERZ11806303).

## References

[1] Garbe C, Buttner P, Bertz J, Stroebel W (1995). Primary cutaneous melanoma: Prognostic classification of anatomic location. Cancer.

[2] Dabouz F (2015). Clinical and histological features of head and neck melanoma: A population-based study in France. Br. J. Dermatol..

[3] Lachiewicz AM, Berwick M, Wiggins CL, Thomas NE (2008). Survival differences between patients with scalp or neck melanoma and those with melanoma of other sites in the surveillance, epidemiology, and end results (SEER) program. Arch. Dermatol..

[4] Xie C (2017). Scalp melanoma: Distinctive high risk clinical and histological features. Australas. J. Dermatol..

[5] Urist MM (1984). Head and neck melanoma in 534 clinical Stage I patients: A prognostic factors analysis and results of surgical treatment. Ann. Surg..

[6] de Giorgi V (2012). The prognostic impact of the anatomical sites in the ‘head and neck melanoma’: Scalp versus face and neck. Melanoma Res..

[7] Tseng WH, Martinez SR (2011). Tumor location predicts survival in cutaneous head and neck melanoma. J. Surg. Res..

[8] Huismans AM (2014). Primary melanoma location on the scalp is an important risk factor for brain metastasis: A study of 1687 patients with cutaneous head and neck melanomas. Ann. Surg. Oncol..

[9] Tas F, Erturk K (2017). Scalp melanoma is associated with high mitotic rate and is a poor prognostic factor for recurrence and outcome. Melanoma Res..

[10] Claeson M (2020). Clinicopathological factors associated with death from thin (≤ 1·00 mm) melanoma. Br. J. Dermatol..

[11] Shumate CR, Carlson GW, Giacco GG, Guinee VF, Byers RM (1991). The prognostic implications of location for scalp melanoma. Am. J. Surg..

[12] Benmeir P (1995). Melanoma of the scalp. Plast. Reconstr. Surg..

[13] Pereira AR (2021). Melanomas of the scalp: Is hair coverage preventing early diagnosis?. Int. J. Dermatol..

[14] Pasquali S (2015). Lymphatic and blood vasculature in primary cutaneous melanomas of the scalp and neck. Head Neck.

[15] Fadaki N (2013). Is head and neck melanoma different from trunk and extremity melanomas with respect to sentinel lymph node status and clinical outcome?. Ann. Surg. Oncol..

[16] Pannucci CJ, Collar RM, Johnson TM, Bradford CR, Rees RS (2012). The role of full-thickness scalp resection for management of primary scalp melanoma. Ann. Plast. Surg..

[17] Cancer T, Atlas G, Atlas G, Office P (2016). HHS public access. Dep. Heal. Hum. Serv..

[18] Tonella L (2021). Prognostic and predictive biomarkers in stage iii melanoma: Current insights and clinical implications. Int. J. Mol. Sci..

[19] Menzies AM (2012). Distinguishing clinicopathologic features of patients with V600E and V600K BRAF-mutant metastatic melanoma. Clin. Cancer Res..

[20] Cirenajwis H (2017). NF1-mutated melanoma tumors harbor distinct clinical and biological characteristics. Mol. Oncol..

[21] Vestergaard ME, Macaskill P, Holt PE, Menzies SW (2008). Dermoscopy compared with naked eye examination for the diagnosis of primary melanoma: A meta-analysis of studies performed in a clinical setting. Br. J. Dermatol..

[22] Zalaudek I (2004). Dermoscopic features of melanoma on the scalp. J. Am. Acad. Dermatol..

[23] Torres F (2010). Dermoscopy of scalp melanoma: Report of three cases. Cancers.

[24] Stanganelli I (2012). Dermoscopy of scalp tumours: A multi-centre study conducted by the international dermoscopy society. J. Eur. Acad. Dermatol. Venereol..

[25] Jaimes N, Marghoob AA (2013). The morphologic universe of melanoma. Dermatol. Clin..

[26] Jaimes N (2015). Clinical and dermoscopic characteristics of melanomas on nonfacial chronically sun-damaged skin. J. Am. Acad. Dermatol..

[27] Hofmann-Wellenhof R (2013). Special criteria for special locations 2: Scalp, mucosal, and milk line. Dermatol. Clin..

[28] Hu R (2015). Trichoscopic findings of androgenetic alopecia and their association with disease severity. J. Dermatol..

[29] Landi MT (2006). MC1R germline variants confer risk for BRAF-mutant melanoma. Science.

[30] Berra CM (2019). Use of uracil-DNA glycosylase enzyme to reduce DNA-related artifacts from formalin-fixed and paraffin-embedded tissues in diagnostic routine. Appl. Cancer Res..

[31] Licata G (2021). Diagnosis and management of melanoma of the scalp: A review of the literature. Clin. Cosmet. Investig. Dermatol..

[32] World Health Organization. WHO. Available at: https://www.who.int.

[33] Benati E (2017). Baldness and scalp melanoma. J. Eur. Acad. Dermatol. Venereol..

[34] Terakedis BE (2014). Patterns of failure and predictors of outcome in cutaneous malignant melanoma of the scalp. J. Am. Acad. Dermatol..

[35] Li W-Q, Cho E, Han J, Weinstock MA, Qureshi AA (2016). Male pattern baldness and risk of incident skin cancer in a cohort of men. Int. J. Cancer.

[36] Porto AC (2020). Primary cutaneous melanoma of the scalp: Patterns of clinical, histological and epidemiological characteristics in Brazil. PLoS ONE.

[37] Richard MA (1999). Melanoma and tumor thickness: Challenges of early diagnosis. Arch. Dermatol..

[38] Cicchiello M, Lin MJ, Pan Y, McLean C, Kelly JW (2016). An assessment of clinical pathways and missed opportunities for the diagnosis of nodular melanoma versus superficial spreading melanoma. Australas. J. Dermatol..

[39] Kalkhoran S (2010). Historical, clinical, and dermoscopic characteristics of thin nodular melanoma. Arch. Dermatol..

[40] Chen LL, Jaimes N, Barker CA, Busam KJ, Marghoob AA (2013). Desmoplastic melanoma: A review. J. Am. Acad. Dermatol..

[41] Xu Z, Yibulayin F, Shi P, Feng L (2018). Desmoplastic melanoma versus spindle cell melanoma: Incidence and survival 1973 to 2017. Medicine.

[42] Quaglino P (2021). Treatment of advanced metastatic melanoma. Dermatol. Pract. Concept..

[43] Bastian BC (2014). The molecular pathology of melanoma: An integrated taxonomy of melanocytic neoplasia. Annu. Rev. Pathol..

[44] Sakaizawa K (2015). Clinical characteristics associated with BRAF, NRAS and KIT mutations in Japanese melanoma patients. J. Dermatol. Sci..

[45] Pracht M (2015). Prognostic and predictive values of oncogenic BRAF, NRAS, c-KIT and MITF in cutaneous and mucous melanoma. J. Eur. Acad. Dermatol. Venereol..

[46] van Kempen LC, Redpath M, Robert C, Spatz A (2014). Molecular pathology of cutaneous melanoma. Melanoma Manag..

[47] Bradish JR, Cheng L (2014). Molecular pathology of malignant melanoma: Changing the clinical practice paradigm toward a personalized approach. Hum. Pathol..

[48] Kiuru M, Busam KJ (2017). The NF1 gene in tumor syndromes and melanoma. Lab. Invest..

[49] Garbarino F (2021). Flat scalp melanoma dermoscopic and reflectance confocal microscopy features correspond to histopathologic type and lesion location. J. Eur. Acad. Dermatol. Venereol..

