# Supplementary material for: A cross-sectional study of clinical, dermoscopic, histopathological, and molecular patterns of scalp melanoma in patients with or without androgenetic alopecia
Source: Sci Rep. 2022 Sep 5;12:15096. doi: 10.1038/s41598-022-17108-z (PMC9445057; doi:10.1038/s41598-022-17108-z)
Supplement: Supplementary file 1 — Supplementary Table 1. [file 41598_2022_17108_MOESM1_ESM.docx]

**Supplement 1:** Dermoscopic characteristics of scalp melanoma in relation to presence of histopathological characteristics

|  | **Alopecia** | | **p** | **Breslow thickness** | | **p** | **Elastosis** | | **p** | **Associated nevus** | | **p** | **Mitosis** | | **p** | **Ulceration** | | **p** |
| --- | --- | --- | --- | --- | --- | --- | --- | --- | --- | --- | --- | --- | --- | --- | --- | --- | --- | --- |
|  | **yes** | **no** |  | **<1mm** | **≥ 1mm** |  | **yes** | **No** |  | **yes** | **no** |  | **yes** | **no** |  | **yes** | **no** |  |
|  | **n (%)** | **n(%)** |  | **n (%)** | **n (%)** |  | **n (%)** | **n (%)** |  | **n (%)** | **n (%)** |  | **n (%)** | **n (%)** |  | **n (%)** | **n (%)** |  |
| **Classic dermoscopic pattern** |  | | | | | | | | | | | | | | | | | |
| Atypical network | 7 (38.9) | 12 (38.7) | 0.999^**^ | 17 (47.2) | 2 (15.4) | 0.092^**^ | 9 (39.1) | 9 (42.9) | 0.999^**^ | 6 (54.5) | 13 (34.2) | 0.386^**^ | 16 (42.1) | 3 (27.3) | 0.492^*^ | 0 (0) | 19 (41.3) | 0.273^*^ |
| Negative network | 1 (5.6) | 2 (6.5) | 0.999^*^ | 1 (2.8) | 2 (15.4) | 0.168^*^ | 1 (4.3) | 1 (9.5) | 0.599^*^ | 1 (9.1) | 2 (5.3) | 0.542^*^ | 1 (2.6) | 2 (18.2) | 0.122^*^ | 0 (0) | 3 (6.5) | 0.999^*^ |
| Streaks | 0 (0) | 2 (6.5) | 0.526^*^ | 1 (2.8) | 1 (7.7) | 0.162^*^ | 0 (0) | 2 (9.5) | 0.222^*^ | 1 (9.1) | 1 (2.6) | 0.402^*^ | 0 (0) | 2 (18.2) | 0.047^*^ | 0 (0) | 2 (4.3) | 0.999^*^ |
| Crystalline structures | 3 (16.7) | 4 (12.9) | 0.697^*^ | 5 (13.9) | 2 (15.4) | 0.999^**^ | 5 (21.7) | 2 (9.5) | 0.416^*^ | 1 (9.1) | 6 (15.8) | 0.999^*^ | 5 (13.2) | 2 (18.2) | 0.646^*^ | 1 (33.3) | 6 (13) | 0.377^*^ |
| Atypical dots or globules | 1 (5.6) | 9 (29) | 0.070^*^ | 9 (25) | 1 (7.7) | 0.253^*^ | 1 (4.3) | 8 (38.1) | 0.008^*^ | 2 (18.2) | 8 (21.1) | 0.999^*^ | 7 (18.4) | 3 (27.3) | 0.673^*^ | 0 (0) | 10 (21.7) | 0.999^*^ |
| Off-center blotch | 4 (22.2) | 12 (38.7) | 0.384^**^ | 13 (36.1) | 3 (23.1) | 0.502^*^ | 6 (26.1) | 9 (42.9) | 0.393^**^ | 4 (36.4) | 12 (31.6) | 0.999^*^ | 14 (36.8) | 2 (18.2) | 0.300^*^ | 0 (0) | 16 (34.8) | 0.541^*^ |
| Regression structures | 12 (66.7) | 14 (45.2) | 0.247^**^ | 21 (58.3) | 5 (38.5) | 0.365^**^ | 14 (60.9) | 8 (38.1) | 0.227^**^ | 8 (72.7) | 18 (47.4) | 0.180^*^ | 25 (65.8) | 1 (9.1) | 0.001^*^ | 1 (33.3) | 25 (54.3) | 0.594^*^ |
| Blue-white veil | 2 (11.1) | 5 (16.7) | 0.696^*^ | 4 (11.1) | 4 (30.8) | 0.183^*^ | 5 (21.7) | 3 (14.3) | 0.701^*^ | 1 (9.1) | 7 (18.4) | 0.663^*^ | 4 (10.5) | 4 (36.4) | 0.063^*^ | 1 (33.3) | 7 (15.2) | 0.421^*^ |
| Atypical vessels | 5 (27.8) | 13 (41.9) | 0.494^**^ | 11 (30.6) | 7 (53.8) | 0.184^*^ | 9 (39.1) | 7 (33.3) | 0.932^**^ | 4 (36.4) | 14 (36.8) | 0.999^*^ | 12 (31.6) | 6 (54.5) | 0.300^**^ | 3 (100) | 15 (32.6) | 0.044^*^ |
| Peripheral tan structureless areas | 10 (55.6) | 2 (6.5) | 0.001^*^ | 11 (30.6) | 1 (7.7) | 0.142^*^ | 10 (43.5) | 2 (9.5) | 0.017^*^ | 4 (36.4) | 8 (21.1) | 0.427^*^ | 11 (28.9) | 1 (9.1) | 0.252^*^ | 0 (0) | 12 (26.1) | 0.566^*^ |
| **Photodamage dermoscopic pattern** |  | | | | | | | | | | | | | | | | | |
| Perifollicular granularity | 11 (61.1) | 8 (25.8) | 0.032^**^ | 16 (44.4) | 3 (23.1) | 0.306^**^ | 14 (60.9) | 4 (19) | 0.007^*^ | 4 (36.4) | 15 (39.5) | 0.999^*^ | 18 (47.4) | 1 (9.1) | 0.033^*^ | 1 (33.3) | 18 (39.1) | 0.999^*^ |
| Asymmetric gray perifollicular openings | 8 (44.4) | 2 (6.5) | 0.003^*^ | 8 (22.2) | 2 (15.4) | 0.709^*^ | 8 (34.8) | 1 (4.8) | 0.023^*^ | 3 (27.3) | 7 (18.4) | 0.673^*^ | 10 (26.3) | 0 (0) | 0.090^*^ | 0 (0) | 10 (21.7) | 0.999^*^ |
| Polygonal structures | 4 (22.2) | 1 (3.2) | 0.054^*^ | 5 (13.9) | 0 (5) | 0.306^*^ | 5 (21.7) | 0 (0) | 0.050^*^ | 2 (18.2) | 3 (7.9) | 0.311^*^ | 5 (13.2) | 0 (0) | 0.574^*^ | 0 (0) | 5 (10.9) | 0.999^*^ |
| Rhomboidal structures | 4 (22.2) | 5 (16.1) | 0.708^*^ | 7 (19.4) | 2 (15.4) | 0.999^*^ | 6 (26.1) | 1 (4.8) | 0.097^*^ | 2 (18.2) | 7 (18.4) | 0.999^*^ | 8 (21.1) | 1 (9.1) | 0.662^*^ | 1 (33.3) | 8 (17.4) | 0.464^*^ |
| Follicle obliteration | 9 (50) | 8 (25.8) | 0.160^**^ | 14 (38.9) | 3 (23.1) | 0.498^*^ | 13 (56.5) | 1 (4.8) | 0.001^*^ | 3 (27.3) | 14 (36.8) | 0.725^*^ | 16 (42.1) | 1 (9.1) | 0.071^*^ | 0 (0) | 17 (37) | 0.542^*^ |
| Circle within a circle | 4 (22.2) | 1 (3.2) | 0.054^*^ | 4 (11.1) | 1 (7.7) | 0.999^*^ | 4 (17.4) | 0 (0) | 0.109^*^ | 1 (9.1) | 4 (10.5) | 0.999^*^ | 5 (13.2) | 0 (0) | 0.574^*^ | 0 (0) | 5 (10.9) | 0.999^*^ |
| Angulated lines | 4 (22.2) | 0 (0) | 0.014^*^ | 4 (11.1) | 0 (0) | 0.562^*^ | 4 (17.4) | 0 (0) | 0.109^*^ | 1 (9.1) | 3 (7.9) | 0.999^*^ | 4 (10.5) | 0 (0) | 0.562^*^ | 0 (0) | 4 (8.7) | 0.999^*^ |
| Patchy peripheral pigmented islands | 1 (5.6) | 0 (0) | 0.367^*^ | 1 (2.8) | 0 (0) | 0.999^*^ | 1 (4.3) | 0 (0) | 0.999^*^ | 0 (0) | 1 (2.6) | 0.999^*^ | 1 (2.6) | 0 (0) | 0.999^*^ | 0 (0) | 1 (2.2) | 0.999^*^ |
| Tan structureless and granularity pattern | 3 (16.7) | 3 (9.7) | 0.656^*^ | 5 (13.9) | 1 (7.7) | 0.999^*^ | 4 (17.4) | 1 (4.8) | 0.348^*^ | 2 (18.2) | 4 (10.5) | 0.605^*^ | 5 (13.2) | 1 (9.1) | 0.999^*^ | 1 (33.3) | 5 (10.9) | 0.330^*^ |
| Diffuse / multifocal hypopigmentation | 11 (61.1) | 8 (25.8) | 0.032^**^ | 16 (44.4) | 3 (23.1) | 0.306^**^ | 11 (47.8) | 6 (28.6) | 0.317^**^ | 6 (54.5) | 13 (34.2) | 0.386^**^ | 17 (44.7) | 2 (18.2) | 0.165^*^ | 0 (0) | 19 (41.3) | 0.273^*^ |

*Fisher’s exact test; ** Chi-square test
