# Supplementary material for: A cross-sectional study of clinical, dermoscopic, histopathological, and molecular patterns of scalp melanoma in patients with or without androgenetic alopecia
Source: Sci Rep. 2022 Sep 5;12:15096. doi: 10.1038/s41598-022-17108-z (PMC9445057; doi:10.1038/s41598-022-17108-z)
Supplement: Supplementary file 2 — Supplementary Table 2. [file 41598_2022_17108_MOESM2_ESM.docx]

**Supplement 2** Estimate of negative binomial generalized linear model (GLM) parameters for the photodamage dermoscopic pattern.

| **Variable** | **Simple GLM** | | | | | | |
| --- | --- | --- | --- | --- | --- | --- | --- |
|  | **Category** | **Coef. (B)** | **SE** | **Exp(B)** | **95% CI** | | **p** |
|  |  |  |  |  | **Lower** | **Upper** |  |
| Alopecia | yes | 1.038 | 0.327 | 2.823 | 1.487 | 5.357 | 0.002 |
|  | no | Ref |  |  |  |  |  |
| Ulceration | yes | -0.693 | 0.836 | 0.500 | 0.097 | 2.575 | 0.407 |
|  | no | Ref |  |  |  |  |  |
| Nevus | yes | 0.155 | 0.416 | 1.168 | 0.517 | 2.639 | 0.709 |
|  | no | Ref |  |  |  |  |  |
| Perineural invasion | yes | -0.672 | 1.425 | 0.511 | 0.031 | 8.343 | 0.637 |
|  | no |  |  |  |  |  |  |
| Sex | female | -0.896 | 0.477 | 0.408 | 0.160 | 1.040 | 0.060 |
|  | male | Ref |  |  |  |  |  |
| Mitosis | >0 | -1.457 | 0.493 | 0.233 | 0.089 | 0.612 | 0.003 |
|  | 0 | Ref |  |  |  |  |  |
| Age (years) | Continue | 0.038 | 0.011 | 1.038 | 1.016 | 1.062 | 0.001 |
| Breslow thickness (mm) | Contínue | -0.202 | 0.103 | 0.817 | 0.668 | 0.999 | 0.049 |
| *BRAF* V600K | yes | 0.116 | 0.532 | 1.123 | 0.396 | 3.186 | 0.827 |
|  | no | Ref |  |  |  |  |  |
| *BRAF* V600E | yes | -0.503 | 0.684 | 0.605 | 0.158 | 2.310 | 0.462 |
|  | no | Ref |  |  |  |  |  |
| *KIT* | yes | 0.453 | 0.820 | 1.574 | 0.315 | 7.854 | 0.580 |
|  | no | Ref |  |  |  |  |  |
| *NF1* | yes | -0.863 | 0.626 | 0.422 | 0.124 | 1.438 | 0.168 |
|  | no | Ref |  |  |  |  |  |
| *NRAS* | yes | 0.255 | 0.842 | 1.290 | 0.248 | 6.720 | 0.762 |
|  | no | Ref |  |  |  |  |  |
|  |  |  |  |  |  |  |  |
|  |  |  |  |  |  |  |  |

SE: Standard error; CI confidence interval;
